# Concerning Wells

**Published:** 1874-10

**Authors:** 


### CONCERNING WELLS.

Something should be let down into wells and vaults before a person descends; if the flame goes out then there is so much carbonic acid gas at the bottom that a person going down would be suffocated. In case a person faints under such circumstances, the most instantaneous relief is to pour down whole tub fulls of cold water. This absorbs the gas, and the man revives; wetting the clothing must not be regarded for an instant. Fresh slaked lime or whitewash will do the same thing; if no one is in the well pour it down; if there is an occupant, let down a vessel of it and keep stirring it with a long pole. Setting straw or shavings on fire, and throwing them in the well while blazing, or letting them down half way with a rope while on fire, creates a draught towards the mouth of the well, and thus empties it of its poisonous air. These things should be impressed on the minds of all families. In the autumn many wells which supply families with drinking and cooking water get very low, and their bottoms are covered with a fine mud, largely the result of vegetable decomposition, also containing poisonous matters of a very concentrated character; the very emanations from this well mud are capable of causing malignant fevers in a few hours; hence many families dependent on well water are made sick during the fall of the year by drinking these impregnated poisons, and introducing them directly into the circulation. Many obscure ailments and "dumb agues" are caused in this way. The remedy is, draw all the water out; scrape the bottom of the well of all its sediment, then wash it most lavishly with cold water, until it seems clear. In this simple way all who depend on wells for water will prevent a great deal of sickness in their families.

